# Molecular Mechanisms of Renal Cellular Nephrotoxicity due to Radiocontrast Media

**DOI:** 10.1155/2014/249810

**Published:** 2014-03-18

**Authors:** Ashour Michael, Teresa Faga, Antonio Pisani, Eleonora Riccio, Placido Bramanti, Massimo Sabbatini, Michele Navarra, Michele Andreucci

**Affiliations:** ^1^Department of Health Sciences, Nephrology Unit, “Magna Graecia” University, I-88100 Catanzaro, Italy; ^2^Department of Nephrology, “Federico II” University, I-80131 Naples, Italy; ^3^IRCCS Centro Neurolesi “Bonino Pulejo”, I-98124 Messina, Italy; ^4^Department of Drug Sciences and Health Products, University of Messina, I-98168 Messina, Italy

## Abstract

Modern iodinated radiocontrast media are all based on the triiodinated benzene ring with various chemical modifications having been made over the last few decades in order to reduce their toxicity. However, CIN remains a problem especially in patients with pre-existing renal failure. *In vitro* studies have demonstrated that all RCM are cytotoxic. RCM administration *in vivo* may lead to a decrease in renal medullary oxygenation leading to the generation of reactive oxygen species that may cause harmful effects to renal tissue. In addition, endothelin and adenosine release and decreased nitric oxide levels may worsen the hypoxic milieu. *In vitro* cell culture studies together with sparse *in vivo* rat model data have shown that important cell signalling pathways are affected by RCM. In particular, the prosurvival and proproliferative kinases Akt and ERK1/2 have been shown to be dephosphorylated (deactivated), whilst proinflammatory/cell death molecules such as the p38 and JNK kinases and the transcription factor NF-**κ**B may be activated by RCM, accompanied by activation of apoptotic mediators such as caspases. Increasing our knowledge of the mechanisms of RCM action may help to develop future therapies for CIN.

## 1. Introduction

Radiocontrast media (RCM) are commonly used in medical practice, but their use may lead to contrast-induced nephropathy (CIN). The continued growth in radiographic examinations means that increasing numbers of patients are exposed to RCM, which in turn has resulted in increasing incidence of CIN. CIN is the third most common cause of hospital-acquired kidney failure accounting for 12% of all cases [1, 2]. Whilst the toxicity of RCM is not fully understood, it is believed to be due to many factors, the two possible principal mechanisms being their effects on renal hemodynamics and direct toxicity on renal cells [3, 4].

The opacity of iodine to X-rays renders it a suitable compound as a contrast medium. However, due to its toxicity, iodine is not suitable to be used in its molecular or ionic form. Whilst many iodinated organic compounds (with covalently bound iodine within the molecule) had been proposed, the search was for a compound that was less toxic, more soluble, and having more opacity (i.e. containing more iodine atoms per molecule). It was in the 1950s that certain derivatives of iodinated benzoic acid were suggested as possible safe contrast media [5], and since then, all modern iodinated contrast media are based on the triiodinated benzene ring. The ratio of iodine atoms to dissolved particles is important since a greater number of iodine atoms would give better opacification and fewer particles in solution would result in a lower osmotic effect, and bearing these characteristics in mind, the evolution of the RCM have involved successive chemical modifications. Firstly, hydrogen atoms on the benzene ring were substituted with acetamido groups to reduce protein binding (protein binding was believed to cause anaphylactoid reactions) giving rise to the acetrizoates and diatrizoates. These compounds are ionic and dissociate in solution and are termed as high-osmolar contrast media (HOCM). Then the carboxyl groups were replaced by nonpolar groups giving nonionic soluble molecules with lower osmolality. These were termed as the low-osmolar contrast media (LOCM) and were further improved by the addition of more hydroxyl groups for increased hydrophilicity and then a more even distribution of the hydroxyl groups on the molecule. Finally, the dimerization of two molecules via side chains on the benzene ring gave rise to the nonionic iso-osmolar contrast media (IOCM) with increased iodine atoms per molecule [6]. Of interest is the RCM ioxaglate that whilst it is ionic, it is classified as an LOCM as it is a dimer containing more iodine atoms per particle in solution. A summary of the most commonly used RCM are presented in Table 1. The improvements in contrast media development have resulted in the acknowledgement that, in clinical use, the newer LOCM and ICOM are less toxic than the original HOCM [7], but* in vitro* cell culture studies have suggested that all types of RCM have a direct toxic effect in many different types of cells (see Table 2). It has been suggested that molecular iodine may be present in solutions of RCM due to degradation [8], but this may be negligible in solutions that have been properly stored. Moreover, a study has shown that incubating isolated proximal tubule segments with varying concentrations of NaI had no adverse effects on cell viability [9], and a further study showed that sodium iodide alone did not cause significant cell death in cultured renal cells [10].

## 2. Toxicity of Radiocontrast Media in* In Vitro* Cell Culture Studies

Many* in vitro* studies have investigated the toxicity of RCM using different types of cultured cells, including renal epithelial cells, mesangial cells, endothelial cells, smooth muscle cells, hepatic cells, human fibroblasts, pulmonary mast cells, human embryonic kidney cells, and human neutrophils.

The most common* in vitro* studies addressing the pathophysiology of RCM-induced apoptosis have been criticized because of their limitations which include: (1) the assessment of only one potential mechanism of the RCM-induced renal cell damage in the absence of several conflicting variables that can be found* in vivo*; (2) the exposure to a constant concentration of RCM to all cell lines, whereas* in vivo*, the more distal epithelial tubular cells are exposed to a much higher concentration than the proximal tubular cells; (3) the potentially high dose of RCM commonly used for the cell culture experiments; (4) the fact that effects of the RCM are usually investigated as cellular “short term”-induced effects (i.e. just after their exposure to cells), not along several hours (as “long term” effects) of exposure to them. Finally, research groups rarely compare different types of RCM (especially those with different osmolarities) in the same study.

The most commonly used types of renal tubular cells for* in vitro* studies include the canine-derived MDCK cells (a model of distal tubular cells), the porcine cell line LLC-PK1 (a model of proximal tubular cells), and the human HK-2 cell line. The last one is a commonly used immortalized human proximal tubular cell line which retains the phenotypic expression and functional characteristic of human proximal tubular cells, as described by others [29, 30].

Different measures of cellular functional/structural changes have been used to indicate cell toxicity due to RCM as outlined in the Table 2.

## 3. Radiocontrast Agents Cause Renal Hypoxia-Role of Reactive Oxygen Species

Many studies have reported that administration of radiocontrast agents causes a decrease in renal medullary oxygenation [31]. This may be due to mechanical factors such as increased blood viscosity (in part related to red blood cell aggregation) and urine viscosity as well as changes in the levels of vasoactive mediators such as endothelins, natriuretic peptides, nitric oxide, adenosine, and prostaglandins [31]. It has also been proposed that the medullary hypoperfusion is caused by constriction of the descending vasa recta (DVR) due to cytotoxic damage of the endothelial cells of the DVR caused by RCM [32]. Using isolated perfused human and rat DVR [32], it was observed that the IOCM iodixanol at physiologically relevant concentrations caused constriction of DVR and caused structural damage of endothelial cells from rat renal interlobular arteries. Thus, it is possible that such RCM-induced effects lead to reduced medullary blood flow in the kidney. A decrease in blood flow and hence in oxygen supply may lead to perturbations in the mitochondrial electron transport chain leading to the production of reactive oxygen species (ROS) that may have a detrimental effect within the cell by oxidizing membrane lipids, inactivating proteins, oxidizing DNA, and activating cell signalling pathways leading to inflammation and cell death [33, 34].* In vitro* studies have suggested that RCM may also lead to ROS production. Sendeski et al., using isolated single specimens of rat descending vasa recta (DVR), demonstrated that iodixanol caused vasoconstriction of the DVR, and the use of the superoxide dismutase (SOD) mimetic Tempol reduced this iodixanol-induced vasoconstriction [35]. In addition to demonstrating that iodixanol causes structural damage to endothelial cells from isolated arteries, the same group has demonstrated that iodixanol caused an increased permeability of HUVEC (human umbilical vein endothelial cell) monolayers and an increased phosphorylation of myosin light chain, an indicator of endothelial cell retraction and increased permeability [32]. Hence, it is feasible that RCM may penetrate through the cell membrane and once in the cytosol may also inflict similar damage to intracellular organelles. Indeed, plasma membrane damage (measured as loss of the membrane proteins caveolin and NaK-ATPase) and mitochondrial damage (cytochrome c release) by ioversol has been reported [9]. As mentioned earlier, disruption of mitochondria may lead to the production of ROS and this may be how RCM can induce the formation of ROS* in vitro* without the need for hypoxia [34, 36]. Zager et al. [9] questioned the role of oxidative stress in RCM renal tubular and cell injury. They found that plasma membrane damage to proximal tubule segments isolated from mice and subjected to the LOCM, ioversol, was not due to oxidant stress since no lipid peroxidation of the tubules was observed. Furthermore, using HK-2 cells, Zager et al. [9] observed that the antioxidant N-acetylcysteine (NAC) failed to protect against RCM toxicity as assessed by lactate dehydrogenase release and MTT reduction.

However, more recent work using a recombinant manganese superoxide dismutase (SOD) administered* in vivo* to rats undergoing diatrizoate treatment caused an improvement in the glomerular filtration rate and a reduction in renal histologic damage [37]. But the use of antioxidants as therapeutic agents for the alleviation of CIN has yielded conflicting data. The use of the antioxidant NAC had been suggested as a means of reducing CIN in patients [38], but successive trials have been contradictory [39], and recently the Acetylcysteine for Contrast-induced nephropathy Trial (ACT) has concluded that NAC does not reduce the risk of CIN [40]. Other antioxidants have been reported to be effective. In a clinical trial, administration of ascorbic acid (vitamin C) protected against CIN, whilst in an animal model rats fed with doses of alpha-tocopherol (vitamin E) before iopromide injection showed decreased tubular injury due to the RCM, increased SOD levels, and reduced malondialdehyde levels [41]. Furthermore, rats fed with the grape seed proanthocyanidin extract and treated with the HOCM diatrizoate showed decreases in biochemical markers of oxidative stress, apoptosis, and renal tissue damage caused by the RCM. Hizoh and Haller [20] observed a protective effect by taurine on DNA fragmentation induced by RCM in MDCK cells but not by NAC. Nonetheless, it should be mentioned that intracellular peroxide levels in cultured glomerular mesangial cells increased upon exposure to diatrizoate and iohexol, and those levels were attenuated by alpha-tocopherol in diatrizoate-treated but not in iohexol-treated cells [42]. Nonetheless, it has been argued that the oxidative stress observed with the use of RCM may be a consequence of the toxicity of the RCM rather than the cause [8].

## 4. RCM Effects on Endothelin Release

RCM have been reported to induce the release of the potent vasoconstrictor peptide endothelin (ET) both* in vivo* and* in vitro*, as well as upregulating ET mRNA transcription [43–45] and also mediating the upregulation of the renal medullary endothelin converting enzyme-1 expression and synthesis [46]. It is believed that the ET-A receptor is involved in vasoconstriction, whilst stimulation of the ET-B receptor has the opposite effect. A study in humans receiving RCM in which both ET receptors were blocked, resulted in a higher incidence of CIN in patients receiving the blocker than those receiving a placebo [47]. However, the use of a specific ET-A antagonist gave a more positive outcome in an* in vivo* rat study, but this was explained by inhibitory effects of the antagonist on tubular transport mechanisms, thereby decreasing the oxygen demand and reducing hypoxia [48].

## 5. Role of Adenosine in CIN

Adenosine is a product of ATP degradation and may arise under the hypoxic conditions arising from RCM administration. Whilst it may cause vasodilatation in most vessels, adenosine causes vasoconstriction in the renal vasculature [49], thereby worsening the hypoxic conditions in the kidney parenchyma. Hence, unselective (theophylline) and adenosine A1-receptor selective antagonists have been used to prevent CIN with some reported positive outcomes [50, 51].* In vitro* mitochondrial damage by RCM may lead to disruption of the electron transport chain which, as in hypoxic conditions, will lead to ATP degradation and adenosine production. The adenosine produced may act as a substrate for xanthine oxidase leading to production of the ROS, hydrogen peroxide, which would be harmful [52]. However, whilst it has been observed that RCM can increase adenosine levels threefold in cultured HK-2 cells, inhibition of xanthine oxidase with oxypurinol did not confer protection [9].

## 6. RCM May Impair Nitric Oxide (NO) Production

In a study by Ribeiro et al. [22], using cultured smooth muscle cells obtained from rat renal artery, HOCM and LOCM but not IOCM were found to lower NO levels. Thus, in this case RCM may act to block a vasodilatory pathway. It has been suggested that one mechanism by which NO levels are lowered is by reaction with superoxide ions that are generated in the kidney by the RCM [53] which would lead to the formation of the even more potent oxidant peroxynitrite anion [54].

## 7. Cellular Signalling Pathways Affected by RCM

Over the course of the last two decades, our knowledge of signal transduction pathways by which cells respond to changes in their environment has increased immensely. These intracellular signalling pathways may determine cell fate, for example, death, survival, proliferation, and release of hormones, and may be triggered by mechanical, chemical, light, and thermal stimuli.

### 7.1. Pathways Involved in Cell Survival and Proliferation

Saito et al. [55] reported a reduction in cyclic adenosine monophosphate (cAMP) levels in mast cells. This was quickly followed by reports of the LOCM ioversol inducing apoptosis in LLC-PK1 cells [21]. Incubation of LLC-PK1 cells with ioversol for 30 minutes followed by a further incubation for 24 h in the absence of the RCM caused an increase in the activities of caspases-3 and -9 (proteases involved in apoptosis) and an increase in the mRNA for the proapoptotic protein Bax, whilst the mRNA levels of the antiapoptotic Bcl-2 decreased. It was also found that use of the cAMP analogue dibutyl(DB)cAMP reversed the changes in a way that was dependent on the signaling molecules protein kinase A (PKA) and phosphatidylinositol 3-kinase (PI 3-K). The same group also found that the prostaglandin I2 analogue beraprost sodium could also reverse the effects of ioversol on caspases and Bax/Bcl-2 [56] by phosphorylation of the cAMP-responsive element binding protein (CREB) via a PKA-dependent mechanism. Further studies, again using LLC-PK1 cells, suggested that the prosurvival kinase Akt may also be involved in the effects of beraprost sodium [57]. Work from our laboratory has shown that incubation of HK-2 cells with an HOCM (sodium diatrizoate), LOCM (iopromide and iomeprol), and IOCM (iodixanol) causes the dephosphorylation of Akt at the Serine473 and Threonine308 sites [24, 26]. This was accompanied by effects on downstream targets of Akt such as p70S6 kinase (inactivated) which is involved in protein synthesis and the FoxO (Forkhead-box) family of transcription factors (dephosphorylated and hence activated) [24]. Both diatrizoate and iopromide caused a decrease in HK-2 cell viability which was partially alleviated by transfection with plasmids encoding constitutively active Akt [24]. Diatrizoate also caused a greater dephosphorylation of mTOR (mammalian target of rapamycin) and ERK1/2 (extracellular signal regulated kinases 1/2) with respect to iopromide, iomerol, or iodixanol [24, 26]. It should also be noted that the dephosphorylation of Akt, p70S6 kinase, and FoxO proteins was observed in primary cultures of human proximal tubule cells [24]. It should be noted that perturbations in the activity of kinases such as the ERK1/2 and Akt may affect the synthesis of vasodilatory and vasoconstrictory molecules. For example, endothelin-1 gene transcription is negatively regulated by Akt and positively regulated by FoxO1 [58], whilst COX-2 expression and prostaglandin F2*α* synthesis may be dependent on ERK1/2 activity [59]. Akt has also been implicated in vasorelaxation [60]. Western blot analysis of whole lysates prepared from kidneys removed from rats treated with the LOCM iomeprol and the IOCM iodixanol also showed lowered levels of phospho-Akt (pAkt) and phospho-ERK1/2 (pERK1/2) compared with lysates obtained from control nontreated rats [26]. An earlier study using the LOCM ioversol also indicated that RCM may cause a decrease in the basal levels of pAkt in mouse kidneys [61]. These authors suggested that RCM enhance* de novo* ceramide synthesis and cause the activation of protein phosphatase 2A (PP2A) which in turn can dephosphorylate Akt. However, PP2A may also act as an ERK- and JNK-phosphatase [62], but our* in vitro* results show discrepancy between the effects of RCM on the phosphorylation status of Akt, ERK1/2, and the JNK (c-jun N-terminal kinase) family of mitogen activated protein kinases (MAPKs). Nonetheless, Itoh et al. also found that use of ceramide synthase inhibitors attenuated renal tubular cell injury induced by ioversol in LLC-PK1 cells and reduced the decreased pAkt levels* in vivo*. Our observations of the decrease in pAkt and pERK1/2 by RCM was surprising since RCM have been associated with ROS production and our experience with HK-2 cells is that these kinases are phosphorylated upon stimulation with hydrogen peroxide [63, 64]. A possible explanation for the decrease in phosphorylation of Akt may be due to disruption of cell membrane rafts that organize membrane-associated molecules such as receptors and kinases and regulate cellular signal transduction [65]. It has been proposed that these membrane rafts allow for the close proximity and interaction between Akt and kinases that phosphorylate it, for example, PDK-1 (phosphoinositide-dependent kinase-1), and any perturbation of the raft structure may interfere with the phosphorylation of Akt. Given the already mentioned physical impairment of the cell membrane by RCM [9, 32], it is possible that RCM could be affecting signalling pathways in this way.

### 7.2. Pathways Involved in Cell Death and Inflammation

Our group also demonstrated the phosphorylation of the p38 and JNK MAPKs, and of the transcription factor NF-*κ*B (Ser 276) by sodium diatrizoate and iomeprol in HK-2 cells [25]. Sodium diatrizoate had a greater effect than iomeprol (at the same concentration of iodine) on phosphorylation of these molecules, which have been implicated in inflammation and upregulation of the proinflammatory cytokine IL-8 [66, 67]. Incubation of HK-2 cells with HOCM, LOCM, and IOCM at concentrations of 75 and 100 mgI/mL for up to 3 h did not result in caspase-3 cleavage [26]. However, in HK-2 cells that had been previously exposed to diatrizoate for 2-3 h and then incubated for a further 22 h (after removal of the RCM), caspase-3 cleavage was observed [25, 26]. It should be noted that the presence of phosphorylated JNKs was detected in renal tubular epithelial cells collected from urine samples of patients, 24 h and 48 h after RCM administration [68]. A possible role for the transcription factor NF-*κ*B was also suggested by Xu et al. [69] who noted that the DNA-binding activity of NF-*κ*B in rat increased after diatrizoate administration.

In contrast, Romano et al. have demonstrated that both LOCM and IOCM caused a marked increase in caspases-3 and -9 activities and poly(ADP-ribose) polymerase fragmentation in HEK293 cells; no effect was observed by them on caspase-8 and -10, thus indicating that the RCM activated apoptosis mainly through the intrinsic pathway [10]. Both RCM induced an increase in protein expression levels of proapoptotic members of the Bcl2 family, Bim and Bad. They also demonstrated that pretreatment with NAC and ascorbic acid but not with sodium bicarbonate could prevent apoptosis in a dose-dependent fashion.

Using HEK293T cells, Lee et al. [70] analyzed the effects of four different contrast media: ionic high-osmolar diatrizoate, ionic low-osmolar iothalamate, nonionic low-osmolar iohexol, and nonionic iso-osmolar iodixanol. They showed that diatrizoate, iodixanol, and iothalamate, but interestingly not iohexol, induced the expression of ATF-2 (activating transcription factor-2) mRNA and phosphorylation of ATF-2 in HEK293T cells in a time-dependent manner. More apoptotic cells were detected in diatrizoate-treated kidney cells than in the saline injection group. Cell death was significantly increased by knockdown ATF-2 expression in the presence of diatrizoate, indicating a protective role of ATF-2 in contrast media-induced apoptosis. Despite the study's limitations, such as the absence of any control conditions (e.g., the use of hyperosmolar solutions since diatrizoate is a high-osmolar RCM) in assessing the role of hypertonicity in CIN pathogenesis and their choice of cells that did not include an adult human proximal tubule cell line (such as HK-2 cells), the work provided new evidence that iodinated contrast media, with the exception of iohexol (a nonionic LOCM), could activate the JNK/ATF-2 signaling pathways. So, this provided a new insight into the mechanism and a potential way of prevention of CIN, showing a differential activation of ATF-2 by different RCM [70].

Gong et al. studied the apoptotic signaling mechanism in CIN and tested whether the new antioxidant N-acetylcysteine amide (NACA) could prevent it, using the RCM iohexol. In this study [71], LLC-PK1 cells were exposed to iohexol in order to observe their cell death with apoptotic features in a dose- and time-dependent manner; they showed that the initiation of iohexol-induced apoptosis was mediated by upregulation of Bax and downregulation of Bcl-2 and Mcl-1 (another anti-apoptotic protein), which was preceded by p38 MAPK activation and iNOS (inducible nitric oxide synthase) induction. The use of inhibitors of p38 MAPK and iNOS partially abolished iohexol-induced apoptosis. They also found that pretreatment with NACA partially protected cells from iohexol-induced death by reverting the expression of Bcl-2, Mc1-1, and Bax expression through inhibition of p38 MAPK and iNOS pathway. NACA, partially protecting LLC-PK1 cells from iohexol-induced apoptosis by suppression of p38 MAPK activation and iNOS protein expression, was more effective than NAC, a widely used antioxidant compound. The authors claimed that a possible explanation is that NACA has better membrane permeation and could therefore be expected to be even more effective than NAC* in vivo* [71]. However, our study with p38 MAPK inhibition only yielded a small increase in cell viability after exposure of HK-2 cells to diatrizoate [26].

**Figure 1:**
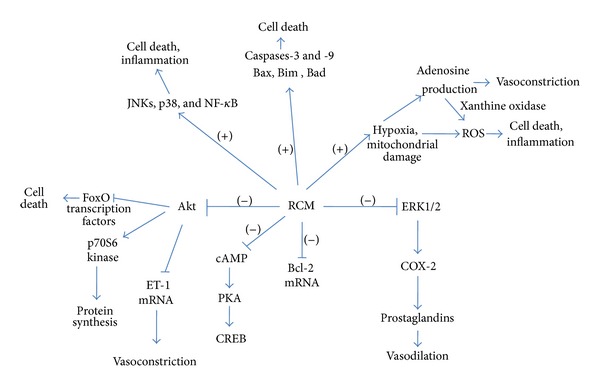
Scheme showing the effects of RCM on signaling molecules. The scheme relates to effects on signaling molecules that may underlie the toxic effects of RCM. RCM cause inactivation of the kinase Akt (as shown by the minus symbol) leading to activation of FoxO transcription factors which may lead to cell death; upregulation of ET-1 transcription and hence vasoconstriction; deactivation of the p70S6 kinase and hence downregulation of protein synthesis. ERK1/2 are also inactivated which may lead to a decrease in activity of COX-2 and prostaglandin production and hence inhibition of vasodilation. RCM also downregulate cAMP and hence the cAMP dependent kinase PKA. The antiapoptotic protein Bcl-2 is downregulated, whilst the proapoptotic proteins Bax, Bim, Bad, and caspases-3 and -9 are upregulated (as shown by the plus symbol) by RCM. The JNK and p38 MAP kinases are activated as also is the transcription factor NF-*κ*B, all three of which may play a role in cell death and inflammation. Hypoxia and mitochondrial damage caused by RCM may lead to the formation of ROS (reactive oxygen species) that can cause cell death and inflammation; and to the formation of adenosine that can cause vasoconstriction, whilst its metabolism by xanthine oxidase can lead to further formation of ROS.

Recently Gong et al. implicated the role of p38 MAPK as well as FoxO1 pathways in RCM toxicity [72]. Using an experimental model of CIN in rats, they showed that tetramethylpyrazine (TMP) could significantly attenuate the resulting renal dysfunction and renal tubular cellular apoptosis. These functional changes were accompanied by the decreased levels of phospho-p38 MAPK protein and attenuation of the increased FoxO1 mRNA and nuclear protein expression [72].  Figure 1 shows a summary of the signaling molecules that play roles in cell death, survival, inflammation and in vasoconstriction/vasodilation, as discussed above.

### 7.3. Cell Survival Pathways Induced by RCM

The cellular stress evoked by RCM has also been shown to prompt an unfolded protein response [UPR] (which is believed to be a prosurvival response) in a rat renal proximal tubular cell line NRK-52E [73]. Wu et al. found that treatment of the cells with diatrizoate caused the expression of the chaperones GRP (Glucose regulated protein) 78 and GRP94 which act to protect the cell under conditions of stress. Furthermore, GRP78 dissociates from the endoplasmic reticulum transmembrane receptor PERK [PKR (double-stranded RNA-activated protein kinase) like ER kinase], and in so doing allowing PERK to be activated. Wu et al. suggested that active PERK phosphorylates and activates the eukaryotic initiation factor (eIF)2*α*, which in turn reduces RCM-induced cell apoptosis [73].

Moon et al. [74] suggested that angiopoietin-1 (Ang1) may protect vascular endothelial cells from iopromide-induced apoptosis through PI 3-K and mTOR/S6 kinase, postulating that the pretreatment with Ang1 could help in maintaining normal vascular endothelial cell integrity before and during systemic RCM administration. In that work Ang1 reduced iopromide-induced apoptosis in a dose-dependent manner. Two PI 3-K inhibitors, wortmannin and LY294002, decreased the Ang1-induced anti-apoptotic effect. Since Ang1 mediates the activation of mTOR/ribosomal protein p70 S6 kinase through PI 3-K, wortmannin, and rapamycin, an inhibitor of mTOR, suppressed Ang1-induced p70S6 kinase phosphorylation and partially inhibited the Ang1-induced anti-apoptotic effect [74].

Yokomaku et al. have suggested that asialoerythropoietin may have potential as a new therapeutic approach to prevent CIN, given its ability to preserve renal function and directly protect renal tissue, as demonstrated in rats, in which nephropathy was induced by injection with the RCM ioversol, in addition to inhibition of prostaglandin and nitric oxide synthesis [75]. The administration of a single dose of asialoerythropoietin before the induction of nephropathy could significantly attenuate the resulting renal dysfunction and the histologic renal tubular injury. RCM-induced apoptosis of renal tubular cells was inhibited by asialoerythropoietin both* in vivo* and* in vitro*, and this effect was blocked by a Janus kinase 2 (JAK2) inhibitor* in vitro*. Furthermore, phospho-JAK2/signal transducer and activator of transcription 5 (STAT5) and heat-shock protein 70 expression increased after injection of asialoerythropoietin, suggesting that the effects of asialoerythropoietin might be mediated by the activation of the JAK2/STAT5 pathway.

Another cellular mechanism that may be protective against RCM injury was reported by Goodman et al. [76]. They demonstrated that heme oxygenase-1 induction by cobalt protoporphyrin could prevent the increase in plasma creatinine and in superoxide ion formation in both the cortex and medulla in uninephrectomized, salt depleted male Sabra rats treated with the RCM sodium iothalamate compared with untreated rats. This protective effect of heme oxygenase-1 was associated with increased anti-apoptotic proteins Bcl-2 and Bcl-xl and with a decrease of proapoptotic caspase-3 and caspase-9 together with increased expression of inactive Bax.

## 8. Conclusions

It is clear that RCM induce a drastic effect both* in vitro* and* in vivo*. Whilst* in vivo* they cause changes in the tissue environment causing hypoxic conditions and changes in levels of vasoconstrictory and vasodilatory factors that may exacerbate the hypoxic milieu,* in vitro* cell culture studies have demonstrated that RCM cause changes in a variety of cell signaling molecules that play important roles in cellular homeostasis. These include the deactivation of molecules such as Akt and ERK1/2 that enable cells to survive stress and to proliferate as well as to regulate the synthesis of vasoactive molecules. At the same time other molecular species are increased or activated that may be detrimental, such as ROS and signalling molecules such as the p38 and JNK kinases and transcription factors such as NF-*κ*B that may mediate cell death and inflammation [77]. Delineation of the molecular mechanisms of RCM may help future strategies to reduce their detrimental effects.

## Figures and Tables

**Table 1:** Iodinated contrast media commonly used in clinical practice.

Name	Type	Iodine content	Osmolality	Osmolality type
		(mg/mL)	(mOsm/kg)	
Ionic				
Diatrizoate (Hypaque 76)	Monomer	370	2,016	HOCM
Metrizoate (Isopaque 370)	Monomer	370	2,100	HOCM
Iothalamate (Conray 400)	Monomer	400	2,300	HOCM
Ioxaglate (Hexabrix)	Dimer	320	580	LOCM
Nonionic				
Iopamidol (Isovue 370)	Monomer	370	796	LOCM
Iohexol (Omnipaque 350)	Monomer	350	884	LOCM
Iopromide (Ultravist 370)	Monomer	370	774	LOCM
Ioversol (Optiray 350)	Monomer	350	792	LOCM
Iomeprol (Iomeron 400)	Monomer	400	720	LOCM
Iobitridol (Xenetix 350)	Monomer	350	915	LOCM
Iodixanol (Visipaque 320)	Dimer	320	290	IOCM
Iotrolan (Isovist 300)	Dimer	300	320	IOCM

Ionic and nonionic contrast media may be monomeric or dimeric; 3 iodine atoms are present on each benzene ring of the contrast medium: if a contrast molecule contains only 1 benzene ring, it is called a monomer, if it contains 2 benzene rings, it is called a dimer. In solution, ionic contrast media break up into their anion and cation components, thereby increasing osmolality, while nonionic contrast media do not break up in solution. Nonionic dimers are the ideal contrast media as they deliver the most iodine with the least effect on osmolality.

The osmolality of contrast media is compared with the osmolality of plasma. HOCM (high-osmolar contrast media) have the highest osmolality, that is, 5–8 times the osmolality of plasma. LOCM (low-osmolar contrast media) have an osmolality still higher than plasma, which is, 2-3 times the osmolality of plasma. IOCM (iso-osmolar contrast media) have the same osmolality as plasma.

**Table 2:** Summary of some *in vitro* cell culture studies using different types of RCM.

Authors/year of publication	Radiocontrast media used; cell type used	Cell functional/structural changes observed
Laerum 1983 [11]	HOCM and LOCM; human endothelial cells	Chromium-51 release as measure of cell toxicity; HOCM more toxic than LOCM

Andersen et al. 1994 [12]	Ionic monomeric/dimeric, nonionic LOCM; MDCK and LLC-PK1 cells	RCM caused formation of large cytoplasmic vacuoles; increase in brush border and lysosomal marker enzyme activity

Dascalu and Peer 1994 [13]	Ionic/nonionic RCM; endothelial and renal cells	Acidification of internal pH; decrease in cell viability

Andersen et al. 1995 [14]	Nonionic LOCM and IOCM; MDCK and LLC-PK1 cells	RCM caused concentration-dependent formation of large cytoplasmic vacuoles; cell death/decrease in cell viability; increase in brush border and lysosomal marker enzyme activity. These effects were more pronounced with LOCM than with IOCM

Potier et al. 1997 [15]	Ionic/nonionic LOCM and HOCM; mesangial cells	Dye uptake as measure of cell viability HOCM more toxic than LOCM

Haller et al. 1997 [16]	HOCM and LOCM; MDCK and LLC-PK1 cells	HOCM more toxic. LLC-PK1 cells more susceptible to RCM cytotoxicity

Hizóh et al. 1998 [17]	MDCK; HOCM	DNA fragmentation caused by RCM

Hardiek et al. 2001 [18]	LLC-PK1 cells and human renal proximal tubule cells; HOCM, LOCM, and IOCM	Cell viability was affected by all RCM with HOCM having a greater affect than IOCM which in turn had a greater effect than LOCM

Fanning et al. 2002 [19]	Human neutrophils; HOCM, LOCM, and IOCM	All types of RCM induced neutrophil apoptosis, with HOCM having greater effect

Hizoh and Haller 2002 [20]	MDCK cells; HOCM	HOCM induced DNA fragmentation

Yano et al. 2003 [21]	LLC-PK1 cells; HOCM and LOCM	HOCM caused a greater decrease in cell viability

Ribeiro et al. 2004 [22]	Renal artery smooth muscle cells; HOCM and LOCM	HOCM caused a greater decrease in cell viability

Heinrich et al. 2005 [23]	LLC-PK1 cells; HOCM, LOCM, and IOCM	All types of RCM cause a decrease in cell viability with HOCM showing greatest effect

Andreucci et al. 2006, 2011, 2014 [24–26]	HK-2 cells; HOCM, LOCM, and IOCM	All RCM caused a decrease in cell viability in the order HOCM > LOCM > IOCM

Heinrich et al. 2007 [27]	LLC-PK1 cells; LOCM and IOCM	Cell viability measured; no difference in toxicity between the 2 types of RCM

Yang et al. 2013 [28]	LOCM (ioversol); rat renal proximal tubular cell line (NRK-52E)	Decrease in cell viability and increase in intracellular Ca^2+^ ion concentration

## References

[1] Nash K, Hafeez A, Hou S (2002). Hospital-acquired renal insufficiency. *American Journal of Kidney Diseases*.

[2] Gleeson TG, Bulugahapitiya S (2004). Contrast-induced nephropathy. *American Journal of Roentgenology*.

[3] Heyman SN, Brezis M, Epstein FH, Spokes K, Silva P, Rosen S (1991). Early renal medullary hypoxic injury from radiocontrast and indomethacin. *Kidney International*.

[4] Humes HD, Hunt DA, White MD (1987). Direct toxic effect of the radiocontrast agent diatrizoate on renal proximal tubule cells. *American Journal of Physiology—Renal Fluid and Electrolyte Physiology*.

[5] Wallingford VH, Decker HG, Kruty M (1952). X-ray contrast media. I. Iodinated acylaminobenzoic acids. *Journal of the American Chemical Society*.

[6] Katzberg RW (1997). Urography into the 21st century: new contrast media, renal handling, imaging characteristics, and nephrotoxicity. *Radiology*.

[7] Morcos SK (2009). Contrast-induced nephropathy: are there differences between low osmolar and iso-osmolar iodinated contrast media?. *Clinical Radiology*.

[8] Sendeski MM (2011). Pathophysiology of renal tissue damage by iodinated contrast media. *Clinical and Experimental Pharmacology and Physiology*.

[9] Zager RA, Johnson ACM, Hanson SY (2003). Radiographic contrast media-induced tubular injury: evaluation of oxidant stress and plasma membrane integrity. *Kidney International*.

[10] Romano G, Briguori C, Quintavalle C (2008). Contrast agents and renal cell apoptosis. *European Heart Journal*.

[11] Laerum F (1983). Acute damage to human endothelial cells by brief exposure to contrast media in vitro. *Radiology*.

[12] Andersen K-J, Christensen EI, Vik H (1994). Effects of iodinated x-ray contrast media on renal epithelial cells in culture. *Investigative Radiology*.

[13] Dascalu A, Peer A (1994). Effects of radiologic contrast media on human endothelial and kidney cell lines: intracellular pH and cytotoxicity. *Academic Radiology*.

[14] Andersen KJ, Vik H, Eikesdal HP, Christensen EI (1995). Effects of contrast media on renal epithelial cells in culture. *Acta Radiologica. Supplementum*.

[15] Potier M, Lagroye I, Lakhdar B, Cambar J, Idee J (1997). Comparative cytotoxicity of low- and high-osmolar contrast media to human fibroblasts and rat mesangial cells in culture. *Investigative Radiology*.

[16] Haller C, Schick CS, Zorn M, Kübier W (1997). Cytotoxicity of radiocontrast agents on polarized renal epithelial cell monolayers. *Cardiovascular Research*.

[17] Hizóh I, Sträter J, Schick CS, Kübier W, Haller C (1998). Radiocontrast-induced DNA fragmentation of renal tubular cells in vitro: role of hypertonicity. *Nephrology Dialysis Transplantation*.

[18] Hardiek K, Katholi RE, Ramkumar V, Deitrick C (2001). Proximal tubule cell response to radiographic contrast media. *American Journal of Physiology—Renal Physiology*.

[19] Fanning NF, Manning BJ, Buckley J, Redmond HP (2002). Iodinated contrast media induce neutrophil apoptosis through a mitochondrial and caspase mediated pathway. *British Journal of Radiology*.

[20] Hizoh I, Haller C (2002). Radiocontrast-induced renal tubular cell apoptosis: hypertonic versus oxidative stress. *Investigative Radiology*.

[21] Yano T, Itoh Y, Sendo T, Kubota T, Oishi R (2003). Cyclic AMP reverses radiocontrast media-induced apoptosis in LLC-PK1 cells by activating a kinase/PI3 kinase. *Kidney International*.

[22] Ribeiro L, de Assunção e Silva F, Kurihara RS, Schor N, Higa EMS (2004). Evaluation of the nitric oxide production in rat renal artery smooth muscle cells culture exposed to radiocontrast agents. *Kidney International*.

[23] Heinrich MC, Kuhlmann MK, Grgic A, Heckmann M, Kramann B, Uder M (2005). Cytotoxic effects of ionic high-osmolar, nonionic monomeric, and nonionic iso-osmolar dimeric iodinated contrast media on renal tubular cells in vitro. *Radiology*.

[24] Andreucci M, Fuiano G, Presta P (2006). Radiocontrast media cause dephosphorylation of Akt and downstream signaling targets in human renal proximal tubular cells. *Biochemical Pharmacology*.

[25] Andreucci M, Lucisano G, Faga T (2011). Differential activation of signaling pathways involved in cell death, survival and inflammation by radiocontrast media in human renal proximal tubular cells. *Toxicological Sciences*.

[26] Andreucci M, Faga T, Russo D (2014). Differential activation of signaling pathways by low-osmolar and iso-osmolar radiocontrast agents in human renal tubular cells. *Journal of Cellular Biochemistry*.

[27] Heinrich M, Scheer M, Heckmann M, Bautz W, Uder M (2007). Reversibility and time-dependency of contrast medium induced inhibition of 3-(4,5-dimethylthiazol-2-yl)-2,5-diphenyl-tetrazolium bromide (MTT) conversion in renal proximal tubular cells in vitro: comparison of a monomeric and a dimeric nonionic iodinated contrast medium. *Investigative Radiology*.

[28] Yang D, Yang D, Jia R, Ding G (2013). Selective inhibition of the reverse mode of Na(+)/Ca(2+) exchanger attenuates contrast-induced cell injury. *American Journal of Nephrology*.

[29] Racusen LC, Monteil C, Sgrignoli A (1997). Cell lines with extended in vitro growth potential from human renal proximal tubule: characterization, response to inducers, and comparison with established cell lines. *Journal of Laboratory and Clinical Medicine*.

[30] Ryan MJ, Johnson G, Kirk J, Fuerstenberg SM, Zager RA, Torok-Storb B (1994). HK-2: an immortalized proximal tubule epithelial cell line from normal adult human kidney. *Kidney International*.

[31] Heyman SN, Rosen S, Rosenberger C (2008). Renal parenchymal hypoxia, hypoxia adaptation, and the pathogenesis of radiocontrast nephropathy. *Clinical Journal of the American Society of Nephrology*.

[32] Sendeski MM, Persson AB, Liu ZZ (2012). Iodinated contrast media cause endothelial damage leading to vasoconstriction of human and rat vasa recta. *American Journal of Physiology—Renal Physiology*.

[33] Li C, Jackson RM (2002). Reactive species mechanisms of cellular hypoxia-reoxygenation injury. *American Journal of Physiology—Cell Physiology*.

[34] Murphy MP (2009). How mitochondria produce reactive oxygen species. *Biochemical Journal*.

[35] Sendeski M, Patzak A, Pallone TL, Cao C, Persson AE, Persson PB (2009). Iodixanol, constriction of medullary descending vasa recta, and risk for contrast medium-induced nephropathy. *Radiology*.

[36] Ozkan G, Ulusoy S, Orem A (2012). Protective effect of the grape seed proanthocyanidin extract in a rat model of contrast-induced nephropathy. *Kidney and Blood Pressure Research*.

[37] Pisani A, Sabbatini M, Riccio E (2013). Effect of a recombinant manganese superoxide dismutase on prevention of contrast-induced acute kidney injury. *Clinical and Experimental Nephrology*.

[38] Tepel M, van der Giet M, Schwarzfeld C, Laufer U, Liermann D, Zidek W (2000). Prevention of radiographic-contrast-agent-induced reductions in renal function by acetylcysteine. *The New England Journal of Medicine*.

[39] Fishbane S (2008). N-acetylcysteine in the prevention of contrast-induced nephropathy. *Clinical Journal of the American Society of Nephrology*.

[40] ACT Investigators (2011). Acetylcysteine for prevention of renal outcomes in patients undergoing coronary and peripheral vascular angiography: main results from the randomized acetylcysteine for contrast-induced nephropathy trial (ACT). *Circulation*.

[41] Kongkham S, Sriwong S, Tasanarong A (2013). Protective effect of alpha tocopherol on contrast-induced nephropathy in rats. *Nefrologia*.

[42] Wasaki M, Sugimoto J, Shirota K (2001). Glucose alters the susceptibility of mesangial cells to contrast media. *Investigative Radiology*.

[43] Heyman SN, Clark BA, Kaiser N (1992). Radiocontrast agents induce endothelin release in vivo and in vitro. *Journal of the American Society of Nephrology*.

[44] Heyman SN, Clark BA, Cantley L (1993). Effects of ioversol versus iothalamate on endothelin release and radiocontrast nephropathy. *Investigative Radiology*.

[45] Sung J-M, Shu GHF, Tsai J-C, Huang J-J (1995). Radiocontrast media induced endothelin-1 mRNA expression and peptide release in porcine aortic endothelial cells. *Journal of the Formosan Medical Association*.

[46] Khamaisi M, Raz I, Shilo V (2008). Diabetes and radiocontrast media increase endothelin converting enzyme-1 in the kidney. *Kidney International*.

[47] Wang A, Holcslaw T, Bashore TM (2000). Exacerbation of radiocontrast nephrotoxicity by endothelin receptor antagonism. *Kidney International*.

[48] Liss P, Carlsson P-O, Nygren A, Palm F, Hansell P (2003). ET-A receptor antagonist BQ123 prevents radiocontrast media-induced renal medullary hypoxia. *Acta Radiologica*.

[49] Hansen PB, Schnermann J (2003). Vasoconstrictor and vasodilator effects of adenosine in the kidney. *American Journal of Physiology—Renal Physiology*.

[50] Erley CM, Duda SH, Schlepckow S (1994). Adenosine antagonist theophylline prevents the reduction of glomerular filtration rate after contrast media application. *Kidney International*.

[51] Katholi RE, Taylor GJ, McCann WP (1995). Nephrotoxicity from contrast media: attenuation with theophylline. *Radiology*.

[52] Wong PCY, Li Z, Guo J, Zhang A (2012). Pathophysiology of contrast-induced nephropathy. *International Journal of Cardiology*.

[53] Cao C, Edwards A, Sendeski M (2010). Intrinsic nitric oxide and superoxide production regulates descending vasa recta contraction. *American Journal of Physiology—Renal Physiology*.

[54] Pacher P, Beckman JS, Liaudet L (2007). Nitric oxide and peroxynitrite in health and disease. *Physiological Reviews*.

[55] Saito M, Itoh Y, Yano T (2003). Roles of intracellular Ca(2+) and cyclic AMP in mast cell histamine release induced by radiographic contrast media. *Naunyn-Schmiedeberg’s Archives of Pharmacology*.

[56] Yano T, Itoh Y, Kubota T, Sendo T, Oishi R (2004). A prostacyclin analog beraprost sodium attenuates radiocontrast media-induced LLC-PK1 cells injury. *Kidney International*.

[57] Yano T, Itoh Y, Kubota T (2005). A prostacyclin analog prevents radiocontrast nephropathy via phosphorylation of cyclic AMP response element binding protein. *The American Journal of Pathology*.

[58] Stow LR, Jacobs ME, Wingo CS, Cain BD (2011). Endothelin-1 gene regulation. *The FASEB Journal*.

[59] Jabbour HN, Sales KJ, Boddy SC, Anderson RA, Williams ARW (2005). A positive feedback loop that regulates cyclooxygenase-2 expression and prostaglandin F2*α* synthesis via the F-series-prostanoid receptor and extracellular signal-regulated kinase 1/2 signaling pathway. *Endocrinology*.

[60] Kobayashi T, Matsumoto T, Kamata K (2005). The PI3-K/Akt pathway: roles related to alterations in vasomotor responses in diabetic models. *Journal of Smooth Muscle Research*.

[61] Itoh Y, Yano T, Sendo T (2006). Involvement of de novo ceramide synthesis in radiocontrast-induced renal tubular cell injury. *Kidney International*.

[62] Liu Q, Hofmann PA (2004). Protein phosphatase 2A-mediated cross-talk between p38 MAPK and ERK in apoptosis of cardiac myocytes. *American Journal of Physiology—Heart and Circulatory Physiology*.

[63] Andreucci M, Faga T, Lucisano G (2010). Mycophenolic acid inhibits the phosphorylation of NF-*κ*B and JNKs and causes a decrease in IL-8 release in H_2_O_2_-treated human renal proximal tubular cells. *Chemico-Biological Interactions*.

[64] Andreucci M, Fuiano G, Presta P (2009). Downregulation of cell survival signalling pathways and increased cell damage in hydrogen peroxide-treated human renal proximal tubular cells by alpha-erythropoietin. *Cell Proliferation*.

[65] Calay D, Vind-Kezunovic D, Frankart A, Lambert S, Poumay Y, Gniadecki R (2010). Inhibition of akt signaling by exclusion from lipid rafts in normal and transformed epidermal keratinocytes. *Journal of Investigative Dermatology*.

[66] Kyriakis JM, Avruch J (2012). Mammalian MAPK signal transduction pathways activated by stress and inflammation: a 10-year update. *Physiological Reviews*.

[67] Nowak DE, Tian B, Jamaluddin M (2008). RelA Ser276 phosphorylation is required for activation of a subset of NF-*κ*B-dependent genes by recruiting cyclin-dependent kinase 9/cyclin t1 complexes. *Molecular and Cellular Biology*.

[68] Quintavalle C, Brenca M, de Micco F (2011). In vivo and in vitro assessment of pathways involved in contrast media-induced renal cells apoptosis. *Cell Death and Disease*.

[69] Xu X, Wu T, Ding X, Zhu J, Zou J, He J (2008). The role of nuclear factor-*κ*B in rats of radiocontrast-media-induced nephropathy. *Journal of Biochemical and Molecular Toxicology*.

[70] Lee H, Sheu S, Yen H, Lai W, Chang J (2010). JNK/ATF2 pathway is involved in iodinated contrast media-induced apoptosis. *American Journal of Nephrology*.

[71] Gong X, Celsi G, Carlsson K, Norgren S, Chen M (2010). N-acetylcysteine amide protects renal proximal tubular epithelial cells against iohexol-induced apoptosis by blocking p38 MAPK and iNOS signaling. *American Journal of Nephrology*.

[72] Gong X, Wang Q, Tang X (2013). Tetramethylpyrazine prevents contrast-induced nephropathy by inhibiting p38 MAPK and FoxO1 signaling pathways. *American Journal of Nephrology*.

[73] Wu CT, Sheu ML, Tsai KS, Weng TI, Chiang CK, Liu SH (2010). The role of endoplasmic reticulum stress-related unfolded protein response in the radiocontrast medium-induced renal tubular cell injury. *Toxicological Sciences*.

[74] Moon SO, Kim W, Kim DH (2005). Angiopoietin-1 reduces iopromide-induced endothelial cell apoptosis through activation of phosphatidylinositol 3′-kinase/P70 S6 kinase. *International Journal of Tissue Reactions*.

[75] Yokomaku Y, Sugimoto T, Kume S (2008). Asialoerythropoietin prevents contrast-induced nephropathy. *Journal of the American Society of Nephrology*.

[76] Goodman AI, Olszanecki R, Yang LM (2007). Heme oxygenase-1 protects against radiocontrast-induced acute kidney injury by regulating anti-apoptotic proteins. *Kidney International*.

[77] Andreucci M (2011). Contrast media and nephrotoxicity: a molecular conundrum. *Giornale Italiano di Nefrologia*.

